# Extended Support Within a Person-Centered Practice After Surgery for Patients With Pituitary Tumors: Protocol for a Quasiexperimental Study

**DOI:** 10.2196/17697

**Published:** 2020-07-21

**Authors:** Sofie Jakobsson, Daniel S Olsson, Eva Andersson, Tobias Hallén, David Krabbe, Ann-Charlotte Olofsson, Oskar Ragnarsson, Thomas Skoglund, Gudmundur Johannsson, Eva Jakobsson Ung

**Affiliations:** 1 Institute of Health and Care Sciences Sahlgrenska Academy University of Gothenburg Gothenburg Sweden; 2 Department of Medicine Sahlgrenska University Hospital Region Västra Götaland Gothenburg Sweden; 3 Department of Internal Medicine and Clinical Nutrition Institute of Medicine, Sahlgrenska Academy University of Gothenburg Gothenburg Sweden; 4 Department of Neurosurgery Sahlgrenska University Hospital Region Västra Götaland Gothenburg Sweden; 5 Institute of Neuroscience and Physiology Sahlgrenska Academy University of Gothenburg Gothenburg Sweden; 6 Department of Rehabilitation Medicine Sahlgrenska University Hospital Region Västra Götaland Gothenburg Sweden

**Keywords:** pituitary tumor, person-centered care, clinical pathway, intervention, quasiexperimental

## Abstract

**Background:**

Patients with pituitary tumors often live with lifelong consequences of their disease. Treatment options include surgery, radiotherapy, and medical therapy. Symptoms associated with the tumor or its treatment affect several areas of life. Patients need to adhere to long-term contact with both specialist and general health care providers due to the disease, complex treatments, and associated morbidity. The first year after pituitary surgery constitutes an important time period, with medical evaluations after surgery and decisions on hormonal substitution. The development and evaluation of extended patient support during this time are limited.

**Objective:**

The aim of this study is to evaluate whether support within a person-centered care practice increases wellbeing for patients with pituitary tumors. Our main hypothesis is that the extended support will result in increased psychological wellbeing compared with the support given within standard of care. Secondary objectives are to evaluate whether the extended support, compared with standard care, will result in (1) better health status, (2) less fatigue, (3) higher satisfaction with care, (4) higher self-efficacy, (5) increased person-centered content in care documentation, and (6) sustained patient safety.

**Methods:**

Within a quasiexperimental design, patients diagnosed with a pituitary tumor planned for neurosurgery are consecutively included in a pretest-posttest study performed at a specialist endocrine clinic. The control group receives standard of care after surgery, and the interventional group receives structured patient support for 1 year after surgery based on person-centeredness covering self-management support, accessibility, and continuity. A total of 90 patients are targeted for each group.

**Results:**

Recruitment into the control group was performed between Q3 2015 and Q4 2017. Recruitment into the intervention group started in Q4 2017 and is ongoing until Q4 2020. The study is conducted according to the Declaration of Helsinki, and the protocol has received approval from a regional ethical review board.

**Conclusions:**

This study entails an extensive intervention constructed in collaboration between clinicians, patients, and researchers that acknowledges accessibility, continuity, and self-management support within person-centeredness. The study has the potential to compare standard care to person-centered practice adapted specifically for patients with pituitary tumors and evaluated with a combination of patient-reported outcomes and patient-reported experience measures. Following the results, the person-centered practice may also become a useful model to further develop and explore person-centered care for patients with other rare, lifelong conditions.

**Trial Registration:**

Researchweb.org. https://www.researchweb.org/is/sverige/project/161671

**International Registered Report Identifier (IRRID):**

DERR1-10.2196/17697

## Introduction

### Background

Pituitary tumors occur at any age, but most often occur in persons at the peak of their professional career [1]. The annual incidence of pituitary tumors is approximately 4.0 per 100,000 inhabitants [1-4]. Pituitary tumors can be divided into nonfunctioning tumors (nonfunctioning pituitary adenoma and craniopharyngioma) and hormone-producing adenomas (prolactinomas, Cushing’s disease, and acromegaly). Although they are histologically benign, the tumor itself and its treatment often lead to lifelong hormone deficiencies, obesity, neurocognitive dysfunction, visual field defects, diabetes insipidus, and other adverse effects due to the pituitary gland’s vital regulatory function and its proximity to the hypothalamus and optic chiasm [5,6]. Patients with pituitary tumors therefore have excess morbidity and mortality [1,2,4,7-9]. The tumors are treated with surgery and, in some cases, radiotherapy, while endocrine-active tumors can also be managed with medical therapy [6]. A substantial proportion of patients experience tumor recurrence during their follow-up [10]. Recurrence is associated with excess mortality; disease control is therefore of vital importance for patient outcomes [11].

Pituitary tumors constitute a substantial chronic disease burden for patients, affecting several areas of life [12,13]. Low self-reported health is evident with symptoms such as fatigue, memory and concentration difficulties, sleeping problems, and sexual dysfunction [14,15]. Partners of patients have described a lack of information regarding the disease and its treatment as well as concerns related to changes in relational aspects, social life, and family life [16]. Unemployment is also more common among patients with pituitary disease, and a substantial number of patients report having missed work or not performing to their potential at work due to their illness [17].

The disease, its treatment, and related morbidity necessitate that patients adhere to long-term contact with specialist and general health care providers. There is limited knowledge on how to support patients with pituitary tumors to better cope with their lifelong condition. Andela and colleagues [18] evaluated structured patient and partner group education introduced to patients several years after their diagnosis. The 8-week program showed increased and sustained self-efficacy 6 months after the education program and, to some extent, improved patient mood. However, patients did not report any significant differences in perceived quality of life, symptoms, coping style, or illness perception. A nurse-led, 9-month educational program specifically designed for patients with Cushing’s syndrome showed improvement in health outcomes such as pain, physical activity, and aspects of quality of life [19].

Recent reforms covering health and medical care have highlighted the importance of designing care in collaboration with each patient through participation in decisions, providing explicit information, and determining patients’ preferences and abilities [20,21]. Care based on person-centeredness has been promoted, whereby care providers inquire how patients view their health situation and about their needs, resources, and preferences [22,23]. Person-centeredness focuses on preserving patient autonomy, function, and wellbeing and strives to emphasize patient involvement through equalizing power between health care professionals and patients with the main goal of an enhanced health situation for each patient.

**Figure 1:**
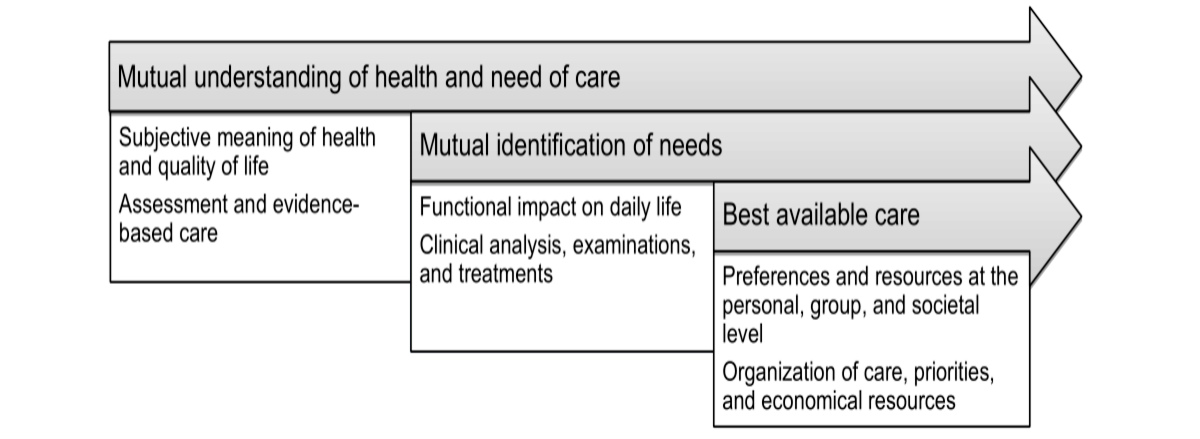
Person-centered practice: integrating person-centered care and the clinical pathway.

This project focuses on how a nurse-led, person-centered practice with a state-of the-art medical team might be beneficial for patients after surgery for pituitary tumors (Figure 1). To our knowledge, this has not been previously studied. This study is presented according to the SPIRIT statement for reporting study protocols [24].

### Study Objectives

The aim of the study is to evaluate whether support within a person-centered care practice increases wellbeing for patients with pituitary tumors. Our main hypothesis is that the extended support will result in increased psychological wellbeing compared with the support given within standard of care. Secondary objectives are to evaluate whether the extended support, compared with standard care, will result in (1) better health status, (2) less fatigue, (3) higher satisfaction with care, (4) higher self-efficacy, (5) increased person-centered content in care documentation, and (6) sustained patient safety.

## Methods

### Study Design

The study utilizes a quasiexperimental design with a nonequivalent control group and a pretest-posttest study design. The study is carried out in two sequential steps: (1) a control group of patients receiving standard care up to 1 year after surgery followed by (2) an interventional group where patients receive extended support within a person-centered practice up to 1 year after surgery.

The study is conducted according to the Declaration of Helsinki. All participants in the study have rights to confidentiality and provide written informed consent [25]. Approval for the protocol has been obtained from the regional ethical review board (approval reference 387-15). Any modifications to study procedures have to be reported as formal amendments to the ethical review board for approval.

### Study Setting

A university clinic in western Sweden constitutes the clinical setting for the study. An inpatient neurosurgical unit, an inpatient endocrine unit following surgery, and an outpatient endocrine unit collectively comprise the patients’ care pathway before and after surgery.

### Study Population

All consecutive patients diagnosed with a pituitary tumor planned for neurosurgery at the study center will be asked to participate in the study. Inclusion criteria are planned neurosurgery and ≥18 years of age. Exclusion criteria comprise health conditions that might restrict understanding of the study or ability to adhere to the protocol (eg, cognitive impairments or drug addiction). The power calculation for estimation of sample size was based on two previous hormonal replacement therapy interventions evaluating improvement in psychological general wellbeing [26,27]. We used a range of d values due to different study designs; based on d=0.3326 (no treatment vs treatment in a crossover design) [27] to d=2.826 (before and after intervention) [26], 64-100 patients are needed in each group to obtain an 80% power with a 95% significance. Considering the different design of the study, 90 patients are targeted in each group, also taking into account a 10% discontinuation rate.

### Recruitment

Recruitment is coordinated by a research nurse (ACO) experienced in the care of patients with pituitary tumors and specifically trained in endocrine research studies. Patients who are planned for surgery and are eligible for the study will be contacted by phone about 1 week before surgery to receive information regarding the study. A written description of the study is sent to the patients after this initial contact. The same nurse meets the patient on the day before surgery to inform about the study again. After answering any potential questions regarding the study, patients provide written informed consent. In the case of acute surgery, the neurosurgeon responsible for the patient obtains verbal and written consent before surgery. No study activities are initiated before verbal and written informed consent.

### Patient and Public Involvement

To be able to develop a valid person-centered practice from the patients’ point of view, a group of patients with pituitary tumors who have experienced long-term care participated in the development of the intervention. In two workshops, discussions were held between patients, clinicians, and researchers on current care and specific needs stated by the patients. The expressed preferences in care were integrated into the content of the intervention. Specifically, these preferences included increased accessibility, an identified contact person within care, patient education program early after surgery, relatives included in care, and knowledge of medical treatment, surgery, and tumor recurrence. When results from the study are assessable, efforts will be made to disseminate the results in appropriate forums for patients and their relatives. In addition, the findings of the study will be communicated in peer-reviewed publications in scientific journals, in PhD theses, and at scientific meetings.

### Intervention

**Figure 2:**
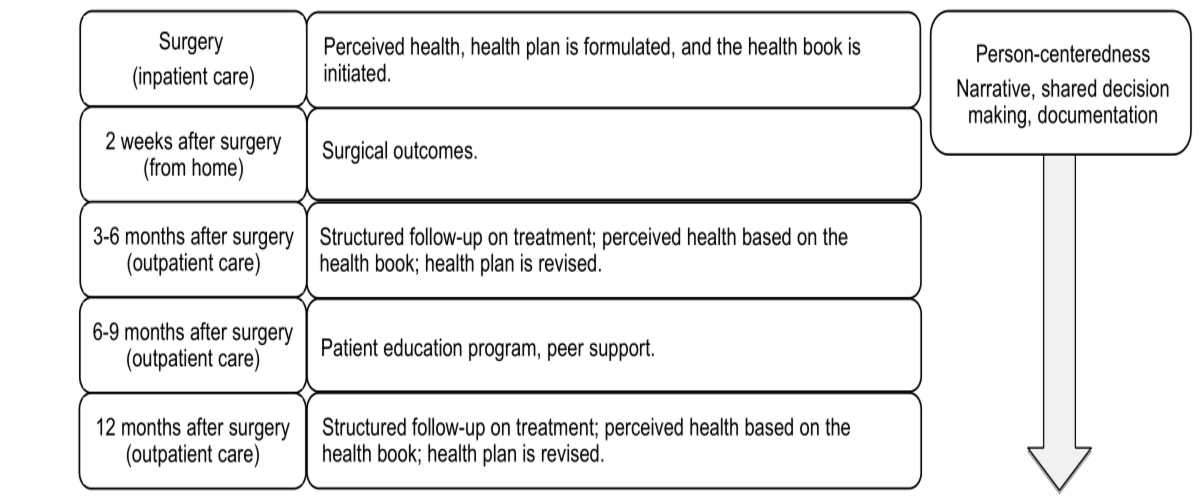
Content of the extended support within a person-centered practice until 1 year after surgery.

The structure and content of the intervention are built on principles for person-centeredness. Within the intervention, repeated patient narratives and continuously revised, documented health care planning ensure that the care is systematically practiced according to principles for person-centeredness (Figure 2). Self-management support*,* being an important component in the intervention, is primarily conducted between the patient and a nurse care manager. Each patient in the intervention is allocated a hospital-initiated nurse care manager, who initiates the first contact with the patient at the inpatient unit before discharge after surgery. The primary goal of the support from the nurse care manager is to facilitate the patient’s own resources in managing illness as well as giving specific health education on, for example, physical activity and diet. Patient-held documentation, the health book, frames the content of self-management support. The health book includes detailed information on the structured clinical care pathway, contact information for the nurse care manager, and preparatory questions that could be used before appointments with the health care team. Questions to encourage reflections on good and bad days and what constitutes or hinders good health are also included in the health book. After each contact between the patient and nurse care manager, a health plan, including agreed goals, is revised. Within the health book, it is also possible for the patient to self-assess and monitor symptoms and health in writing or by drawing. They are also able to reflect and self-assess issues related to work, social support, economy, and other health factors such as physical activity, sleep, sex life, and diet.

**Figure 3:**
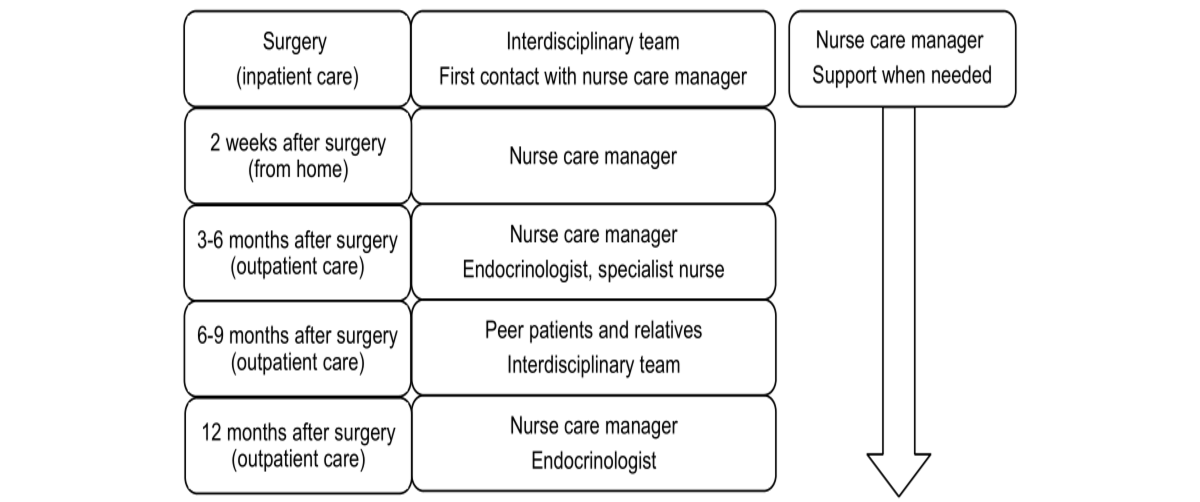
Care contacts in the structured clinical care pathway from surgery until 1 year after surgery. The interdisciplinary team includes endocrinologists, neurosurgeons, nurses, dietitians, and physical therapists.

Other components of the intervention comprise accessibility and continuity*,* which are secured by a structured clinical care pathway (Figure 3). The patient has continuous access to the nurse care manager by telephone and face-to-face contact according to a structured follow-up plan for 1 year after surgery. An interdisciplinary team as well as a patient education program constitute distinct parts of support. The structured clinical care pathway visualizes the care that is preplanned and defines the roles of the different heath care providers. The patient education program at 6-9 months after surgery comprises education for both the patient and their relatives. It is aimed at promoting the development of skills and knowledge needed to self-manage health. The content includes information about surgical treatment, symptoms, and signs as well as issues related to health and quality of life. The patients’ knowledge of diagnosis and surgery is increased by lectures. Discussions during the program are targeted at including common experiences and skills needed to manage different symptoms in daily life. The patient education program also provides requisites for peer support in nonclinical aspects.

To qualify as an outpatient nurse care manager, the nurse must have experience caring for patients with pituitary tumors. Further, it is mandatory to attend a 1-day education course covering updated information on signs and symptoms, hormonal treatments, neurosurgical aspects, person-centered care, health promotion, and the role of the nurse care manager. Each nurse, depending on their experience and needs, completes observation in different care units such as neurosurgery or inpatient care or with specific team members, such as dietitians and physical therapists, included in the patient care pathway. Literature and other materials are made available to the nurse care managers.

### Standard Care

All patients in the control group will receive standard care. Standard care is primarily hospital-based. In short, during the first 24 postoperative hours, the patients are monitored at a medium care unit for neurological status as well as fluid and electrolyte balance. Thereafter, monitoring for a further 5 days is conducted at the inpatient department of endocrinology. Before discharge, on postoperative day 6, evaluation of endocrine deficiencies is performed. At an outpatient visit at the department of endocrinology, 4-5 weeks postoperatively, hormonal status is rechecked, and information on any complications is collected. Thereafter, the frequency of visits to the outpatient clinic depends on tumor types, surgical outcome, hypopituitarism, and perceived symptoms.

### Strategies to Secure the Intervention Over Time

To facilitate and secure the implementation of the intervention over time, and specifically the nurse care manager’s role, the nurse assigned for quality and safety improvement at the clinic (EA) and a researcher from the research group (EJU) meet the nurse care managers regularly to support and discuss the different components of the intervention and its implementation in patient care. In addition, all staff members at the inpatient and outpatient units are informed about the study prior to starting. Specific nurses at the postsurgical endocrinology unit were identified and are involved in the performance of the study; these nurses also attend the 1-day education course. Every 3 months, the nurse assigned for quality and safety improvement at the clinic and the researcher from the research group meet with inpatient and outpatient team members including nurses, endocrinologists, neurosurgeons, research nurses, the head of nurses, and the head of physicians to provide an update on the intervention and discuss issues related to the structured clinical care pathway and other issues of relevance.

### Outcome Measures

The primary outcome measure is self-reported psychological wellbeing. Secondary outcome measures will be self-reported satisfaction of care, health status, fatigue, and self-efficacy. Additional secondary outcomes include person-centered content in documentation and patient safety.

### Participant Timeline

**Figure 4:**
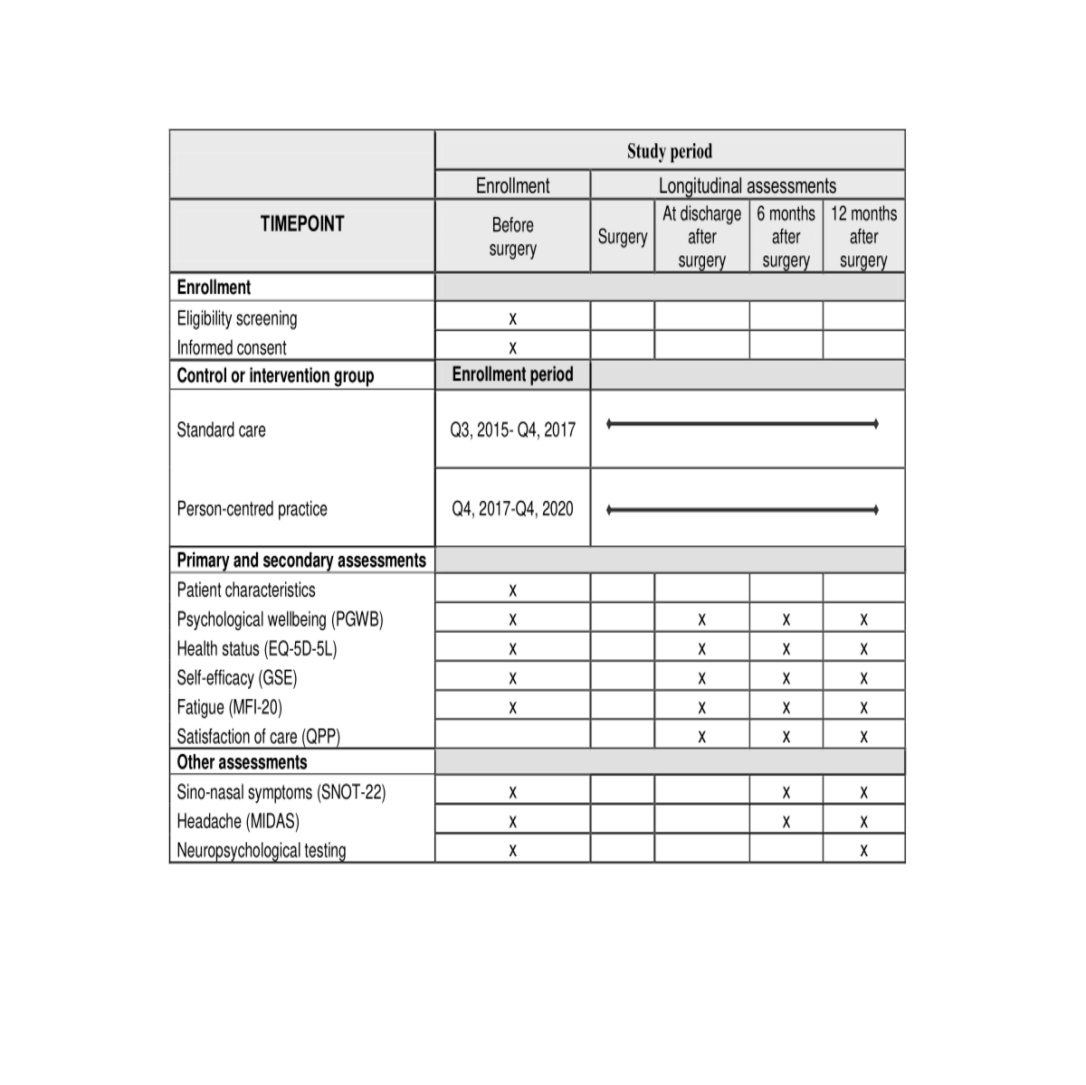
Schedule of enrollment, intervention and assessments for each included patient. EQ-5D-5L: Euro- Qual Five Dimensions; GSE: Generalized Self-Efficacy Scale; MFI-20: The Multidimensional Fatigue Inventory; MIDAS: The Migraine Disability Assessments; PGWB: The Psychological General Well-being instrument; QPP: Quality from the Patient Perspective Questionnaire; SNOT-22: Sino-nasal Outcome Test.

The patient-reported outcome measures are reported by the patients on the day before surgery and repeated at discharge from inpatient care, approximately 1 week after surgery. Two follow-ups are performed at 4-6 months and 11-13 months after surgery (Figure 4). All questionnaires for self-assessment were chosen due to their widespread use in research and good psychometric validity in both patients and healthy populations. Medical records are also reviewed.

### Data Collection

#### Primary Outcome

Self-perceived psychological wellbeing is assessed with the Psychological General Well-Being scale, a 22-item questionnaire comprising 6 dimensions: anxiety, depression, positive wellbeing, self-control, general health, and vitality [28]. The Swedish version of the questionnaire has been externally validated [29]. A total score of 132 is calculated from 6-point Likert scales for each item and represents excellent psychological wellbeing.

#### Secondary Outcomes

The Euro-Qual Five Dimensions questionnaire is used to assess self-reported health status. It includes 5 specific dimensions of health: mobility, self-care, usual activities, pain/discomfort, and anxiety/depression [30-32]. The questionnaire also comprises a visual analogue scale from 0 to 100 in which the patient rates health from the worst to the best health imagined. To further explore effects on the patients’ perceived perception and intensity of fatigue, the Multidimensional Fatigue Inventory-20 is used [33,34]. Within the Multidimensional Fatigue Inventory-20, general fatigue, physical fatigue, reduced activity, reduced motivation, and mental fatigue represent the 5 dimensions of fatigue. The Generalized Self-Efficacy scale is used to measure how the patient perceives their possibility of adhering to the goals being set and find solutions to unforeseen or surprising situations and challenges [35]. As a patient-experience measure, the Quality from the Patient's Perspective questionnaire is used [36,37]. Patients rate their experience and subjective importance in aspects of care: medical-technical competence, identity-oriented approach, sociocultural atmosphere, and physical-technical conditions. Documentation held by both health care professionals (eg, medical records) and patients (eg, the health book) are reviewed with respect to person-centered content [38]. Aspects of patient safety are reviewed from medical records (eg, the assessment of symptoms and vital signs) and care planning as well as specific aspects of care following surgery and introduction of hormonal replacement therapy.

### Retention and Data Management

The research nurse is responsible to schedule follow-up visits in the longitudinal assessment. The research nurse ensures that the scheduled appointments are kept and that data are collected. Reasons for missing data or discontinuation from the study are documented. Updated information on recruitment and follow-up are continuously discussed within the research group to identify structural barriers for inclusion or data collection. A file for data collection on the patients including their social security number and assigned code number is kept in a locked safe at the clinic. Self-reported measures are primarily collected through electronic devices. Data are entered with the code number assigned to each patient. Clinical data such as diagnosis, surgery, medical treatment, radiotherapy, mortality, morbidity, care contacts, and hospitalization are collected from the patients' medical records and entered into data files. Researchers within the research group are responsible for monitoring data collection.

### Plan for Statistical Analysis

Statistical analysis will include descriptive analysis and comparisons between the control and intervention groups. For numerical data, mean, SD, median, and interquartile range will be calculated. Categorical data will be expressed as proportions (%). Background and clinical characteristics for the two groups will be expressed with descriptive data, and differences will be analyzed with the chi‐squared test, independent samples *t* test, or Mann-Whitney U test. The primary outcome will primarily be evaluated by comparing changes in psychological wellbeing between baseline and 1 year after surgery in the two groups. Parametric or nonparametric tests will be used depending on whether data are normally distributed for the purpose of comparing data between the intervention group and control group. Effects will be described as mean differences with 95% CIs. Data will be analyzed using SPSS software package (IBM Inc, Armonk, NY). The significance level will be set at *P*<.05, and all tests will be two-tailed. Subgroup analyses will be performed based on demographic characteristics and disease and treatment characteristics.

### Ancillary Studies

#### Identification of Factors Predicting a Poor Outcome

Tumor remission is highly dependent on the type and growth pattern of the tumor. Excess mortality is highest in patients with craniopharyngioma and lowest in patients with nonfunctioning pituitary adenoma [1,2]. Further, factors often associated with excess mortality and morbidity in these patients are hypopituitarism, female gender, young age at diagnosis, and tumor characteristics needing additional treatment [1,2,4,7,9]. Patient characteristics, magnetic resonance imaging evaluations of the tumor, laboratory results, and tumor tissues are studied to explore factors that may predict long-term outcomes. The anatomical structures surrounding the tumor (hypothalamus and basal forebrain) are visualized on magnetic resonance imaging using sequences for anatomical imaging (T1) as well as sequences for detecting damage (3D T2/FLAIR). Tumor tissue is analyzed to investigate DNA, RNA, expressed proteins, and DNA methylation pattern. This integrated part of the project enhances knowledge with respect to identification of persons at risk for tumor progression.

#### Brain Injury Biomarkers

Resection of large pituitary tumors may lead to manipulation of adjacent structures such as the hypothalamus and the basal forebrain. In an effort to study possible brain damage caused by surgery, we examine peripheral blood biomarkers of brain injury before surgery, immediately after surgery, and during follow-up. This allows us to study the potential relationship between brain injury markers during surgery and long-term outcomes.

#### Consequences and Complications of Surgical Treatment

Surgery for pituitary tumors is usually performed using a transsphenoidal route, which affects sino-nasal structures and may lead to nasal symptoms postoperatively and impaired quality of life [39]. The aim of this part of the study is to examine postoperative effects focusing on sino-nasal symptoms [40]. As headache can occur both as a consequence of the tumor itself and as a complication after surgery, a further aim is to study the occurrence and type of headache before surgery and during follow-up [41].

#### Cognitive Functioning

For an evaluation of cognitive functioning in conjunction with surgery, cognitive functioning is assessed using the Repeatable Battery for the Assessment of Neuropsychological Status, which measures immediate memory, visuospatial functions, language, attention, and delayed memory [42]. The cognitive testing is performed by a neurorehabilitation psychologist before surgery and 1 year after surgery. Together with self-reported quality of life, cognitive function is an important outcome against which intervention, tumor size, surgery, and brain injury markers can be evaluated.

#### Methodological Development

An additional measure of self-efficacy, the Self-Efficacy Scale for chronic disease [43], is used for psychometric evaluation and compared directly with the Generalized Self-Efficacy scale regarding responsiveness and sensitivity.

## Results

Inclusion in the control group receiving standard care was completed from Q3 2015 to Q4 2017 with the number of patients needed to evaluate the primary outcome. Recruitment to the group exposed to the intervention has been ongoing since Q4 2017, and the estimated timepoint for completed recruitment is Q4 2020. Final data collection is expected in Q4 2021.

## Discussion

The primary aim of this study is to evaluate whether support within a person-centered care practice increases psychological wellbeing for patients with pituitary tumors. The project addresses the effects of extended support from surgery to the start of lifelong endocrine treatment and tumor surveillance for patients with pituitary tumors. The year after pituitary surgery constitutes an important time period, with medical evaluations of surgery and decisions on hormonal substitution, all resulting in a critical time for the patients [44].

Clinical care is currently standardized and evidence-based within the framework of a clinical pathway and is based on the medical needs patients have as a group. While it is important to ensure that patients receive safe, high-quality medical care, the patients’ experiences, will, resources, and motivation often have a smaller role in a standardized care model. In this intervention, the clinical pathway and care based on person-centeredness will be integrated (Figure 1). Person-centered practice may create a better foundation for offering care at the right time with the right effort and at the right level, since the patients’ experiences with their own health situation and confidence in their own ability to cope with the situation are the focus.

Recent research has shown that shared decision making can increase physical and mental wellbeing, self-care, and confidence in one’s own abilities [45]. Ekman and colleagues [46] described three integrated procedures when initiating, integrating, and securing the practice of person-centered care: (1) the narrative that puts the person and his or her health and life situation at the center of care, (2) shared information and shared decision making, and (3) documentation that gives legitimacy to the patient's experiences, preferences, beliefs, and values [46]. After receiving care based on these procedures, patients have reported increased satisfaction with care, increased participation in decisions, and care in accordance with needs [38,47]. Furthermore, there was increased confidence in one’s own abilities and reduced uncertainty [48-50]. Person-centered care has also resulted in reduced durations of inpatient care [51].

The core element of the intervention, a nurse care manager, has the potential to give extended support to the patients. The role of a nurse care manager has mainly evolved within cancer care [52]. The role has changed over recent years from facilitating cancer screening to including provision for education to support informed decision making, assessing and addressing psychosocial needs, and facilitating transitions between care providers [52]. The role of the nurse care manager described within our intervention is to provide self-management support based on patient education, the patients’ own resources, access to the interdisciplinary team, and through peer support. In reviewing 35 studies on nurse-led self-management programs in different long-term conditions, interventions aimed at combining both education and skill advancement in relation to individual needs were the most effective in increasing self-efficacy, coping, and behavioral changes, especially when also involving partners [53]. Educating patients about their disease should be individualized and extended to different ways of teaching [54]. The most effective support to increase patients’ health and quality of life by a nurse care manager is to include education and support for self-care specifically adapted to the patient population and provided within an interdisciplinary team [55].

The quasiexperimental design has the potential to compare person-centered practice to standard care. A design based on randomization of parallel groups was considered impossible as the intervention consists of reorganizational as well as relational components and would thereby create potential bias in the standard care group. Developing, implementing, and evaluating a person-centered care practice contains several interacting components that make it a complex intervention [56]. Evaluations of conducting person-centered care have shown that interventional studies demanded specific adaptation to the different clinical settings including time, workload, care culture, and documentation systems that would otherwise constrain the intervention unless continued education and follow-up were performed during the intervention [57,58]. The person-centered practice within our study demands a transformation from disease-oriented care to care based on person-centeredness; therefore, extensive implementation of both structural and relational components is addressed. The study protocol has a design in which the evaluation of the intervention is being made within the setting where it is later supposed to work in clinical practice adapted specifically for patients with pituitary tumors. Following our study’s results, the person-centered practice may also become a useful model to further develop and explore person-centered care for patients with other rare, lifelong conditions.
